# Alemtuzumab as graft-versus-host disease (GVHD) prophylaxis strategy in a developing country: lower rate of acute GVHD, increased risk of cytomegalovirus reactivation

**DOI:** 10.1590/1414-431X20165566

**Published:** 2017-02-09

**Authors:** C.B. Resende, B.M. Rezende, P.T.T. Bernardes, G.M. Teixeira, M.M. Teixeira, V. Pinho, H. Bittencourt

**Affiliations:** 1 Laboratório de Resolução da Resposta Inflamatória, Departamento de Morfologia, Instituto de Ciências Biológicas, Universidade Federal de Minas Gerais, Belo Horizonte, MG, Brasil; 2 Unidade de Transplantes e Serviço de Hematologia, Hospital das Clínicas, Universidade Federal de Minas Gerais, Belo Horizonte, MG, Brasil; 3 Laboratório de Imunofarmacologia, Departamento de Bioquímica e Farmacologia, Instituto de Ciências Biológicas, Universidade Federal de Minas Gerais, Belo Horizonte, MG, Brasil; 4 Hematology-Oncology Division, C.H.U. Saint-Justine, University of Montreal, Montreal, QC, Canada

**Keywords:** Hematopoietic stem cell transplantation, Alemtuzumab, Graft-versus-host disease, Cytomegalovirus

## Abstract

Acute graft-versus-host disease (aGVHD) and cytomegalovirus reactivation are important complications after allogeneic stem cell transplantation (alloHSCT). Here, we evaluated the impact of treatment with alemtuzumab on the occurrence of aGVHD, cytomegalovirus reactivation and survival after alloHSCT. This was a prospective cohort study conducted at the allo-HSCT unit of Hospital das Clínicas, Universidade Federal de Minas Gerais, Brazil, from January 2009 to December 2011. Fifty-seven patients who underwent alloHSCT were included. Forty-five (79%) patients had a malignant disease. Alemtuzumab was administered before the conditioning regimen at a dose of 1 mg/kg in children and 30 mg/day for 2 days in adults or children weighing more than 40 kg (a total dose of 60 mg) with a non-malignant disease or patients with a malignant disease and high-risk for GVHD mortality. Alemtuzumab was used in 23 (40%) patients, of whom 17 received a reduced-intensity conditioning. Eleven patients presented aGVHD (grade 2–4) and only 1 of them received alemtuzumab. Cumulative incidence of aGVHD (grade 2–4) at day 100 after transplantation (D+100) was 4 for patients receiving alemtuzumab and 29% for patients not receiving alemtuzumab. Cumulative incidence of cytomegalovirus reactivation for patients receiving or not alemtuzumab was 62 and 38%, respectively. Sixteen patients died in the first 100 days after alloHSCT, most of them due to bacterial sepsis. Only 2 patients died of aGVHD until D+100. Overall survival was 50% without any impact of alemtuzumab. Alemtuzumab effectively controlled aGVHD but increased the risk of cytomegalovirus reactivation without improving survival.

## Introduction

Acute graft-versus-host disease (aGVHD) and infection/reactivation of cytomegalovirus (CMV) are among the most frequent and important complications after allogeneic hematopoietic stem cell transplantation (alloHSCT). Acute GVHD is reported in up to 32% in related alloHSCT and up to 52% in unrelated alloHSCT (1,2). Primary CMV infection occurs in up to 30% of seronegative patients transplanted with a seropositive graft and CMV disease occurs in up to 20% in patients without prophylaxis or preemptive treatment (3,4). CMV pneumonia still results in high mortality after HSCT, but is considered a rare complication (3,5). Many alloHSCT factors, such as graft source, human leucocyte antigen (HLA) compatibility and conditioning regimen may increase the risk of occurrence and the severity of aGVHD and/or CMV infection (6–8).

Alemtuzumab is a monoclonal antibody that recognizes the antigen CD52, a glycoprotein found in various leukocytes, including lymphocytes, monocytes, macrophages and some dendritic cells. It has been increasingly used, especially in low intensity alloHSCT, in order to reduce GVHD and transplant-related mortality (TRM) (9). Alemtuzumab is useful as part of the conditioning regimen due to its capacity to deplete immunocompetent T-cells and for its characteristic long half-life in bloodstream, lasting even for weeks after administration (10,11). Mechanisms involved in alemtuzumab immunosuppression are not fully understood, but one of its well-known effects is the lysis of B and T lymphocytes, via mechanisms including complement system activation, natural killer cells (NK) and macrophages (6,10).

We have previously shown that, using a conventional GVHD prophylaxis with cyclosporine and methotrexate, incidence of grade 2–4 aGVHD was higher than expected in our center and resulted in lower survival (12). In order to reduce incidence of aGVHD and increase survival, we modified our GVHD prophylaxis strategy, and included alemtuzumab for patients with non-malignant diseases, for unrelated alloHSCT and for patients with a higher risk of TRM (i.e., older patients and/or with comorbidities). Thus, the present prospective cohort study aimed at assessing the impact of alemtuzumab as a strategy to decrease acute GVHD incidence and its effect on CMV reactivation and survival of patients undergoing alloHSCT in a developing country.

## Subjects and Methods

### Study design

This was a prospective cohort study, conducted from January 2009 to December 2011 at the allo-HSCT unit of the Divisão de Hematologia, Hospital das Clínicas, Universidade Federal de Minas Gerais (HC-UFMG), a 500-bed general university hospital in Southeast Brazil and a reference for alloHSCT within the public health system. The study population consisted of 57 patients, 34 (60%) males, and 23 (40%) females, with a median age of 24 (2–56) years. Patients were included in the study after signing an informed consent, regardless of age and other factors. All patients were followed-up from the day of transplantation (day 0) until 1 year after alloHSCT, and were monitored for aGVHD and CMV (up to day 100 after transplantation – D+100).

### Definitions and study endpoints

For the purposes of this study, the conditioning regimen was considered myeloablative when a total oral dose of bussulfan (Bu) of more than 10 mg/kg was used alongside with cyclophosphamide. All other conditioning regimen were considered reduced intensity conditioning (RIC; e.g., cyclophosphamide alone, fludarabine and melphalan, etc.).

GVHD prophylaxis included the combination of a calcineurin inhibitor (cyclosporine or tacrolimus) with or without methotrexate (MTX) or mycophenolate mofetil. For patients with a higher risk of GVHD (unrelated donor), higher risk of TRM (older patient or with comorbidities), or with non-malignant conditions (such as severe aplastic anemia), intravenous alemtuzumab was administered immediately before conditioning at a dose of 1 mg/kg in children (under 40 kg) and 30 mg/day for 2 days in adults or children with more than 40 kg (for a total of 60 mg).

High-resolution HLA typification was used and a donor-recipient pair was considered a match with the presence of full compatibility in HLA-A, B, C and DRB1 locus (8/8 match). All donor/recipient pairs not fulfilling these criteria were considered mismatch.

aGVHD was scored according to the previously described grading system (3,4). aGVHD was diagnosed through clinical signs and graded according to the affected organ. Liver, gastro-intestinal tract and skin were all graded 0 to 4 according to the onset of the first signs and the severity of the disease. Biopsy of the affected organ was not systematically performed. Overall grading and association of the organs affected by acute GVHD was conducted using the aGVHD consensus criteria and scored according to the following grades: absent or mild (0 to 1) and moderate to severe (2 to 4) (3,4,6).

CMV monitoring with pp65 antigenemia assay was performed through identification of the pp65 antigen in blood cells, via antigen-antibody reaction and was obtained on a weekly-basis in engrafted patients, under the criteria of platelet count above 50×10^9^/L and/or neutrophil count above 0.5×10^9^/L. More than 1 pp65 positive cell per 100,000 analyzed cells was considered positive. PCR was not performed for CMV infection diagnosis.

### Statistical methods

Incidence rate and frequency of alloHSCT-related variables are reported as number and percentage or median and range. Categorical variables were assessed through chi-square or Fisher's test. Cumulative incidence of acute GVHD incidence, and CMV reactivation with death as a competitive risk were analyzed using the Fine and Grey's test. Overall survival was calculated through Kaplan-Meier estimator and differences between variables were analyzed using the log rank test. Multivariate analyses were performed using Cox proportional hazards regression model for overall survival, and Fine and Gray's proportional hazards regression model for grade 2–4 GVHD, and CMV reactivation. Variables included in the multivariate model included gender, age and use of alemtuzumab. Statistical analyses were carried out using SPSS version 15.0 (SPSS Inc., USA) and EZR Kanda (Japan). P<0.05 was considered to be statistically significant.

## Results

### Patients and transplant characteristics

Patient and transplant characteristics are shown in Table 1. Thirty-eight patients were not included in this study. The main reason for non-inclusion was denial to sign the informed consent (by patients or their parents).

**Table:**
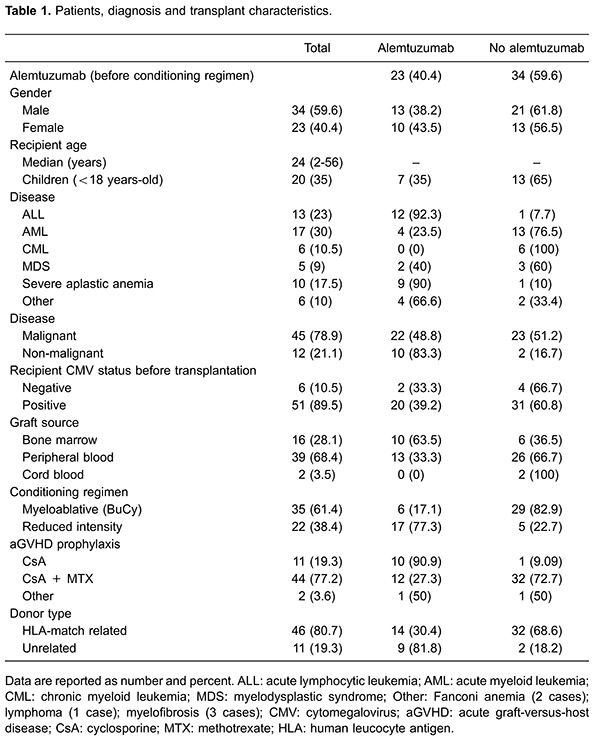

A total of 45 (79%) patients had a malignant disease. Twelve (21%) had non-malignant disorders, mainly severe aplastic anemia that accounted for 18% of cases (10 patients). Median age was 24 (range 2-56) years. The predominant conditioning regimen was the association of myeloablative doses of bussulfan and cyclophosphamide in 35 (61%) patients. Twenty-two (39%) patients underwent a RIC regimen. Alemtuzumab was used before the conditioning regimen in 23 (40%) patients, of whom 17 received RIC conditioning.

GVHD prophylaxis therapy consisted of the combination of cyclosporine and MTX in 44 (77%) patients. Cyclosporine alone was used in 11 (19%) patients.

### Acute GVHD

**Figure 1:**
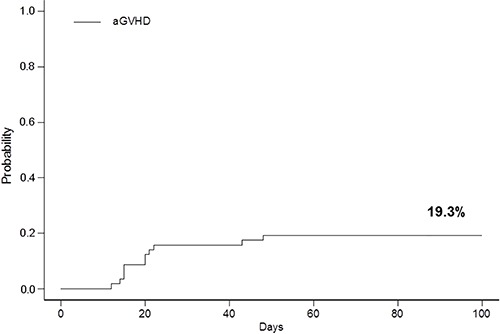
Acute graft-versus-host disease (aGVHD, Grade 2–4) cumulative incidence.

aGVHD occurred in 15 (26%) patients. As expected, most patients had skin and/or gastrointestinal aGVHD. Eleven patients presented grade 2–4 aGVHD. Cumulative incidence rate of grade 2–4 aGVHD was 19% at D+100 (Figure 1).

**Figure 2:**
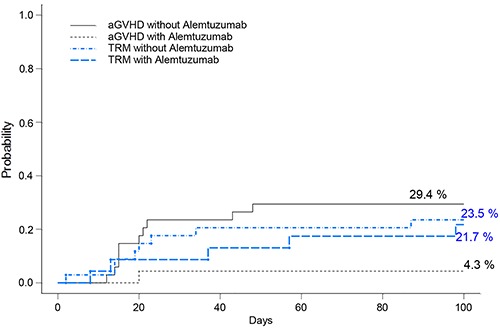
Cumulative incidences of (grade 2–4) acute graft-versus-host disease (aGVHD) and transplant-related mortality (TRM) at day 100 after transplantation for patients receiving or not alemtuzumab before allogeneic stem cell transplantation.

Grade 2–4 aGVHD occurred in only 1 (2%) patient receiving alemtuzumab before the conditioning regimen, and in 10 (18%) patients not receiving alemtuzumab (Table 2). Cumulative incidences of grade 2–4 aGVHD at D+100 for patients receiving or not alemtuzumab before alloHSCT was 4.3 and 29.4%, respectively (P=0.02; Figure 2). In addition, total mortality related to the use of alemtuzumab was 22% (Figure 2). In multivariate analysis, use of alemtuzumab [HR=0.11 (95%CI=0.014-0.878); P=0.04] was associated with a lower incidence of grade 2–4 aGVHD while patient's age [HR: 1.05 (95%CI=1.01-1.10); P=0.03] was associated with an increased risk of grade 2–4 aGVHD.

**Table:**
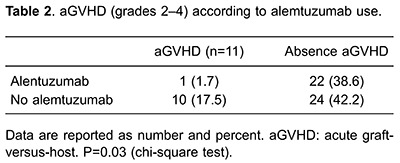

### CMV reactivation

Forty-six (81%) of the 57 patients were tested with the pp65 antigenemia assay. The 11 patients that were not tested include 9 patients that died before engraftment and 2 patients in which the assay was not performed due to technical problems.

**Figure 3:**
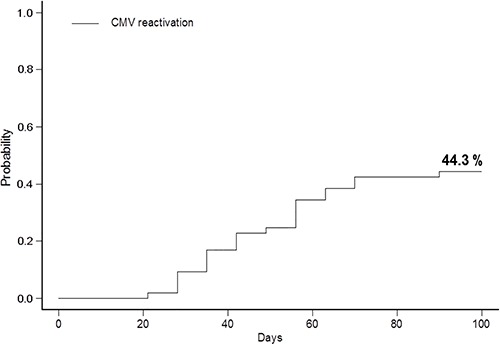
Cumulative incidence of a positive cytomegalovirus (CMV) antigenemia assay at day 100 after transplantation.

**Figure 4:**
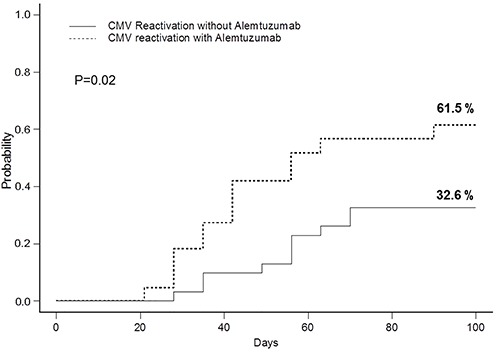
Cumulative incidence of cytomegalovirus (CMV) reactivation at day 100 after transplantation for patients receiving or not alemtuzumab.

Twenty-three (50%) of the 46 tested patients presented a positive antigenemia for CMV. Cumulative incidence of a positive pp65 antigenemia assay after transplant was 44% at D+100 (Figure 3). Use of alemtuzumab before conditioning significantly influenced the incidence of CMV reactivation after alloHSCT. Cumulative incidence of CMV reactivation at D+100 for patients receiving or not alemtuzumab was 62 and 32%, respectively (P=0.02; Figure 4). Use of alemtuzumab [HR: 2.39 (95%CI=1.02-5.60); P=0.04) was the only variable associated with a higher incidence of CMV reactivation in multivariate analysis.

### Transplant-related mortality and survival

**Figure 5:**
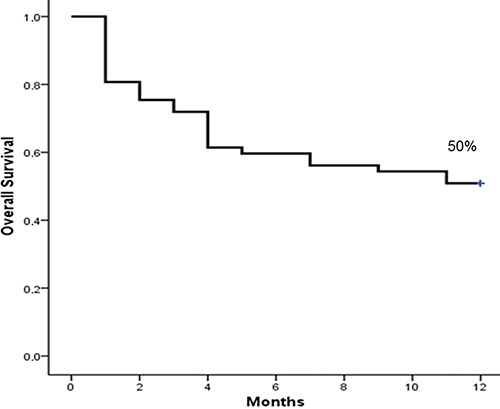
Overall survival at 1 year.

Sixteen patients died in the first 100 days after alloHSCT, of which only in 2 (12%) the cause of death was reported as a result of aGVHD (none receiving alemtuzumab). Nine (56%) deaths were caused by bacterial sepsis. Four of the 5 remaining deaths were caused by other alloHSCT complications and 1 due to relapse of the underlying disease. Overall survival at 1 year was 50% (Figure 5). Alemtuzumab had no impact on survival both in univariate and multivariate analysis.

## Discussion

In the present study, we describe the effects of aGVHD prophylaxis with alemtuzumab in patients undergoing alloHSCT. Our major findings are as follows: 1) the cumulative incidence of aGVHD (grades 2–4) and aGVHD-related deaths were significantly reduced in patients who received alemtuzumab; however, 2) CMV events were significantly higher in patients who received alemtuzumab and alloHSCT, independently of GVHD development; and 3) there was no impact of alemtuzumab on overall survival.

aGVHD and CMV infection/disease are two of the major complications of alloHSCT. aGVHD is the cause of death in up to 14% of unrelated alloHSCT (7) and up to 50% of these patients will die within 2 years of aGVHD diagnosis (13). In the alloHSCT Unit of HC-UFMG, alemtuzumab given before conditioning regime effectively reduced aGVHD (grades 2–4), compared to our historical control (12) and to patients not receiving alemtuzumab. Recent studies show a decrease in aGVHD development risk in patients treated with alemtuzumab when compared to patients receiving antithymocyte globulin (ATG) or standard prophylactic drugs, such as cyclosporine/tacrolimus and MTX (6,10,14–16).

Alemtuzumab is a monoclonal antibody, which recognizes the antigen CD52, a glycosylphosphatidylinositol-anchored glycoprotein present in plasma membrane of T and B lymphocytes, monocytes, thymocytes and macrophages (9). The immunological mechanisms associated with the protective effect of alemtuzumab on acute GVHD remains poorly understood, but it may be associated to depletion of lymphocytes due to complement-mediated lysis and antibody-dependent cellular cytotoxicity via activation of NK cells and macrophages through fragment C antibody (11,17–19). Our data are consistent with previous data and show that treatment with alemtuzumab is effective to prevent aGVHD development and aGVHD-related death in alloHSCT transplanted patients. However, although this treatment appears to control aGVHD, the use of alemtuzumab has been questioned due to an increase in the incidence of CMV infection after alloHSCT. This increase is probably due to delayed immunological reconstitution after transplantation in alemtuzumab-treated patients (6,14,20). Moreover, it is of note that there is a high prevalence of CMV positive serology in Brazil, and the use of drugs that increase the risk of CMV reactivation after alloHSCT, such as alemtuzumab, can pose and extra hazard to patients. To illustrate this point, the prevalence of CMV seropositivity in southern Brazil is 96% in adults (21,22).

Alemtuzumab can be detected in the bloodstream up to 56 days after its administration, resulting in a long period of immunosuppression and susceptibility to infections (10,23). The optimal recommended dose of alemtuzumab for alloHSCT is still a point of discussion. Most studies have used doses superior to the one used in the current study (6,15,24). However, a total dose lower than 60 mg in adults has been recently described to be effective in the control of aGVHD and was associated with improvement in immune reconstitution in patients undergoing unrelated allo-HSCT (16,25). In addition, a lower dose appears to cause less CMV reactivation (26).

In cases of high risk of CMV reactivation, administration of prophylactic antiviral drugs in pre-transplantation has been considered effective to control CMV reactivation (27). However, this strategy is associated with higher cost, late CMV disease and delayed hematopoietic recovery due to antiviral chemotherapy resistance and toxicity (2,4,28). The most commonly used strategy to control CMV reactivation is preemptive treatment that associates monitoring of the virus through antigenemia assay and/or PCR coupled with anti-CMV treatment. These methods allow early identification of infected cells by CMV and guide the use of preemptive treatment with antiviral drugs, such as ganciclovir or foscarnet. Quantitative PCR has been increasingly used instead of antigenemia since it is far more sensitive and does not have the limitation of the number of neutrophils in blood, allowing detection of CMV before engraftment. Unfortunately, in our institution, quantitative PCR is not available as a routine procedure for CMV control after alloHSCT, resulting in delay and/or lack of CMV quantification for patients with long periods of cellular aplasia. Use of quantitative PCR for CMV detection in patients receiving alemtuzumab could improve identification of CMV and allow early preemptive treatment, resulting in less morbidity and mortality.

Our aims for the introduction of alemtuzumab as prophylaxis for patients with high-risk of GVHD/TRM or for those with non-malignant disorders were to decrease the incidence of aGVHD and, consequently, the incidence of TRM, improving overall survival. While the first objective was reached, i.e., significant reduction of aGVHD incidence, there was no impact on overall survival with alemtuzumab treatment. The significant impact of prolonged immunosuppression after alemtuzumab exposure, expressed by the higher rate of CMV reactivation, played a major role on the maintenance of the morbidity and mortality rate in our population. Furthermore, the poor socio-economic conditions of patients and lack of adequate infrastructure at public university hospitals may have increased the risk of infection in this high-risk population and alemtuzumab did not improve this situation.

Our study has some limitations, including the relatively small sample and the relatively elevated number of transplanted patients (40% in the study period) that were not included in this trial. Nevertheless, this is the first study describing the use of alemtuzumab as a preventive measure for aGVHD in Brazil. This prospective cohort shows that alemtuzumab, although effective in the control of aGVHD, did not improve survival. Our results might have an impact on the use of alemtuzumab as part of the GVHD prophylaxis in developing countries. Furthermore, we emphasize the need for effective strategies to prevent and monitor CMV reactivation in patients receiving alemtuzumab after alloHSCT.
